# Analysis of *FOXD3* sequence variation in human ocular disease

**Published:** 2012-06-27

**Authors:** Bethany A. Volkmann Kloss, Linda M. Reis, Dominique Brémond-Gignac, Tom Glaser, Elena V. Semina

**Affiliations:** 1 Department of Pediatrics and Children’s Research Institute at the Medical College of Wisconsin and Children’s Hospital of Wisconsin, Milwaukee, WI; 2 Department of Cell Biology, Neurobiology and Anatomy at the Medical College of Wisconsin, Milwaukee, WI; 3 Department of Pediatric Ophthalmology, St Victor University Hospital of Amiens and Vision Institute, Picardie Jules Verne University, Amiens, France; 4 Departments of Internal Medicine and Human Genetics, University of Michigan, Ann Arbor, MI

## Abstract

**Purpose:**

The migratory neural crest cell population makes a significant contribution to the anterior segment structures of the eye. Consequently, several anterior segment dysgenesis phenotypes are associated with mutations in genes expressed during neural crest development. The forkhead box D3 (*FOXD3*) gene encodes a forkhead transcription factor that plays an important role in neural crest specification in vertebrates and therefore may be involved in human eye disease.

**Methods:**

We screened 310 probands with developmental ocular conditions for variations in *FOXD3*.

**Results:**

Six nonsynonymous *FOXD3* variants were identified. Four of these changes, c.47C>T (p.Thr16Met), c.359C>T (p.Pro120Leu), c.517A>C (p.Asn173His), and c.818_829dup (p.Arg273_Gly276dup), affected conserved regions and were observed primarily in probands with aniridia or Peters anomaly; out of these four variants, one, p.Arg273_Gly276dup, was not detected in control populations and two, p.Pro120Leu and p.Asn173His, were statistically enriched in cases with aniridia or Peters anomaly. The p.Arg273_Gly276dup variant was seen in a proband with aniridia as well as two additional unrelated probands affected with anophthalmia or congenital cataracts. The p.Asn173His variant affects Helix 2 of the DNA-binding domain and was observed in two unrelated patients with Peters anomaly or aniridia; in both cases, one parent carried the same allele.

**Conclusions:**

*FOXD3* variants increase the risk of anterior segment dysgenesis phenotypes in humans. The p.Asn173His mutation affects a residue in the forkhead domain that is 100% conserved among vertebrate orthologs and is predicted to participate in protein–protein interactions. Its phenotypic effects may be modulated by transcriptional cofactors which have yet to be identified.

## Introduction

Abnormal development of the anterior eye leads to a wide spectrum of ocular malformations, which increase glaucoma risk [1]. During embryogenesis, migratory neural crest cells make a significant contribution to the anterior ocular structures, including the cornea, iris and ciliary body [2,3]. Several anterior segment dysgenesis conditions, such as Axenfeld-Rieger and Peters anomalies, are associated with mutations in genes that regulate neural crest cell development (reviewed in [4,5]). The forkhead box D3 (*FOXD3*) gene encodes a forkhead transcription factor with a role in segregation of the neural crest lineage from the neuroepithelium and maintenance of neural crest cells in an undifferentiated state during early stages of patterning by repressing melanogenesis and thus allowing other neural crest derivatives to develop [reviewed in 6]. Mutations in at least 12 forkhead box genes have been implicated in Mendelian disorders: *FOXC1* (OMIM 601090), *FOXC2* (OMIM 602402), *FOXE1* (OMIM 602617), *FOXE3* (OMIM 601094), *FOXF1* (OMIM 601089), *FOXG1* (OMIM 164874), *FOXI1* (OMIM 601093), *FOXL2* (OMIM 605597), *FOXN1* (OMIM 600838), *FOXP1* (OMIM 605515), *FOXP2* (OMIM 605317), and *FOXP3* (OMIM 300292) [7].

The structure and function of *FOXD3* are conserved among vertebrates. In mice, *Foxd3* transcripts are detected in blastocyst stage (E6.5) embryos, throughout the epiblast and in the extraembryonic region [8]. During mid-gestation (E9.5-E10.5), *Foxd3* is expressed in pre-migratory and migratory neural crest cells in the head and tail regions, but expression decreases in differentiated cells derived from the neural crest [9]. While mice with a heterozygous *Foxd3* deletion appear healthy and normal, *Foxd3^−/−^* embryos die shortly after implantation, around E6.5, and show a correlated loss of embryonic (epiblast) cells and expansion of extraembryonic tissues [8]. Conditional deletion of the *Foxd3* coding region in neural crest cells in *Foxd3^flox/–^*; *Wnt1-Cre* mice results in neonatal lethality [10]. By E16.5, embryos with neural crest-specific loss of *Foxd3* have nervous system defects and variable craniofacial malformations, including cleft face and palate, and a subgroup have cardiac defects. In zebrafish, *foxd3* is first expressed during gastrulation, at the neural plate border, and in the tailbud mesoderm, somites, and floor plate [11]. While *foxd3* expression is observed in premigratory neural crest cells, its expression is downregulated as these cells emerge from the dorsal neural tube and differentiate. Some *foxd3* expression, however, persists transiently in a subset of migrating neural crest cells in the somites and peripheral glia [12,13]. Zebrafish deficient in *foxd3* exhibit cardiac and craniofacial defects and embryonic lethality [13-15]. In addition, foxd3 has been shown to negatively regulate the expression of microphthalmia-associated transcription factor a (*mitfa*), which controls cell fate specification of melanocytes in zebrafish; thus, foxd3 prevents neural crest precursors from differentiating into melanophores, instead promoting iridophore specification [16,17]. In chick and *Xenopus*, over- or ectopic expression of *FoxD3* interferes with neural crest differentiation [9,18-20]. Taken together, these data indicate that *FOXD3* is required for development of neural crest derivatives and that its actions are dosage-sensitive.

No mutations have been reported in the human *FOXD3* coding region. However, one study reported an association between vitiligo, an autoimmune skin condition characterized by progressive patchy depigmentation, and the chromosome 1p31 region that includes *FOXD3*, in a multi-generation family with 13 affected individuals [21]. Sequencing of the coding and promoter regions of *FOXD3* and eight other genes identified a heterozygous −639G>T substitution in the *FOXD3* promoter which co-segregated with the disease phenotype in the family and was not seen in matched controls [22]. This substitution increases *FOXD3* transcription, which may interfere with melanoblast differentiation, creating an autoantigen and predisposing to vitiligo.

Given the importance of neural crest cells in the formation of the anterior eye, and the example of *FOXC1* and *FOXE3* mutations in human anterior segment disease [23-27], we screened a cohort of subjects with ocular anomalies for *FOXD3* mutations. We identified four variants affecting conserved regions in five patients with aniridia or Peters anomaly.

## Methods

### Patient samples

Human subjects research approval was obtained from Institutional Review Boards at the Children’s Hospital of Wisconsin, the University of Michigan, and Paris 7 University Hospitals. Written informed consent was provided by all participants and/or their legal guardian, as appropriate. Blood or buccal samples were collected from probands and available family members. DNA was extracted by standard methods.

### Screening of human DNA samples

The full coding region of *FOXD3* was amplified by PCR using four sets of primers (Appendix 1). Thermal cycling conditions for sets 1–3 were performed as follows: 94 °C for 2 min, followed by 38 cycles of 94 °C for 1 min, 60 °C for 1 min, 72 °C for 1.5 min, and a final elongation at 72 °C for 10 min. Thermal cycling conditions for set 4 were performed as follows: 94 °C for 5 min, followed by 38 cycles of 94 °C for 45 s, 60 °C for 45 s, 72 °C for 1 min, and a final elongation at 72 °C for 7 min. Bidirectional sequencing of the PCR products was performed using ABI 3730XL sequencer and protocols (Applied Biosystems/Life Technologies, Carlsbad, CA). Sequencing reactions were performed using the same forward and reverse primers used for amplification, with one exception: set 4 PCR product was sequenced using internal set 4b (Appendix 1). The sequences were analyzed manually and with Mutation Surveyor software (SoftGenetics, State College, PA). Available family members were also screened for *FOXD3* coding changes.

Normal controls included DNA samples from 190 Caucasian, 95 African-American, 95 Asian, and 96 Hispanic individuals. Caucasian controls were obtained from the European Collection of Cell Culture (Salisbury, UK), and other control samples were obtained from the Coriell Institute (Camden, NJ). Additional comparisons were performed using the NHLBI Exome Variant Server (EVS) [28] and the Single Nucleotide Polymorphism (SNP) database [29]. Allele frequencies were calculated based on the number of chromosomes with sufficient quality scores (in-house data) and/or coverage (EVS dat*a*) for each region, then compared between cases and pooled controls by the Fisher's exact test [30].

Paired box gene 6 (*PAX6)* [31,32] screening was performed using eight sets of primers (Appendix 1) and the following cycling conditions: 94 °C for 5 min, followed by 38 cycles of 94 °C for 45 s, 60 °C for 45 s, 72 °C for 1 min, and a final elongation at 72 °C for 7 min. PCR reactions were performed using standard buffers (5 Prime Inc., Gaithersburg, MD) and conditions with DMSO (5%) and betaine (20%) in each reaction. Bidirectional sequencing of the PCR products and sequence analysis was performed as described above. *PAX6* copy number was determined using TaqMan (Applied Biosystems/Life Technologies) probes Hs00255072_cn (overlapping intron 1 and exon 2) and Hs02661628_cn (within the last exon).

Previous genetic screens in the ocular disease cohort included *FOXE3* (forkhead box E3), *CYP1B1* (cytochrome P4501B1), *B3GALTL* (beta-1,3-galactosyltransferase-like), *PITX2* (pituitary paired-like homeodomain transcription factor 2), *FOXC1* (forkhead box C1), and *PITX3* (paired-like homeodomain 3) [33-37].

## Results

### Screening the *FOXD3* coding region

DNA samples from 310 unrelated patients with developmental eye anomalies were screened for variations in the *FOXD3* coding region by PCR sequencing. The primary diagnoses include 79 cases with anophthalmia/microphthalmia, 63 with aniridia, 57 with Peters anomaly, 44 with congenital cataracts, 36 with anterior segment dysgenesis (including Axenfeld-Rieger anomaly) or isolated glaucoma, and 31 with other ocular disorders. Six nonsynonymous variants were identified (Table 1). Four of these changes affected conserved regions and were identified primarily in probands with aniridia or Peters anomaly; out of these four variants, one was not detected in control populations and two were statistically enriched in cases compared to controls.

**Table 1:** *FOXD3* nonsynonymous variants and their distribution in Peters anomaly/aniridia cases and controls.

** **	** **	** **	** **	**Patient alleles**		**Control alleles**		** **
**Allele**	**SNP**	**Protein effect**	**Protein region**	**Distribution**	**Frequency**	**Distribution**	**Frequency**	**Fisher’s exact test**
c.47C>T	rs184767331	p.T16M	NH_2_-terminal	1/232	0.004	0/912^1^	0.0007	p=0.165
** **	** **	** **	** **	** **	** **	8/10552^2^	** **	** **
c.262_273del	rs151026788	p.A88_G91del	NH_2_-terminal	1/232	0.004	5/894^1^	0.006	p=1
** **	** **	** **	** **	** **	** **	N/A^2^	** **	** **
c.286G>T	rs2274188	p.V96L	NH_2_-terminal	5/232	0.036	44/894^1^	0.017	p=0.597
** **	** **	** **	** **	** **	** **	42/4238^2^	** **	** **
c.359C>T*	Not reported	p.P120L*	NH_2_-terminal	1/232	0.004	1/910^1^	0.0001	p=0.045
** **	** **	** **	** **	** **	** **	0/8978^2^	** **	** **
c.517A>C*	rs151021417	p.N173H*	DNA-binding forkhead domain	2/240	0.008	0/946^1^	0.000085	p=0.001
** **	** **	** **	** **	** **	** **	1/10758^2^	** **	** **
c.818_829dup	Not reported	p.R273_G276dup	COOH-terminal	1/238	0.004	0/910^1^	0	p=0.207
** **	** **	** **	** **	** **	** **	N/A^2^	** **	** **

^1^In-house control data; ^2^EVS data; N/A- not applicable (insertions/deletions are not recorded in EVS); * alleles with statistically significant difference in distribution between cases and controls.

**Figure 1:**
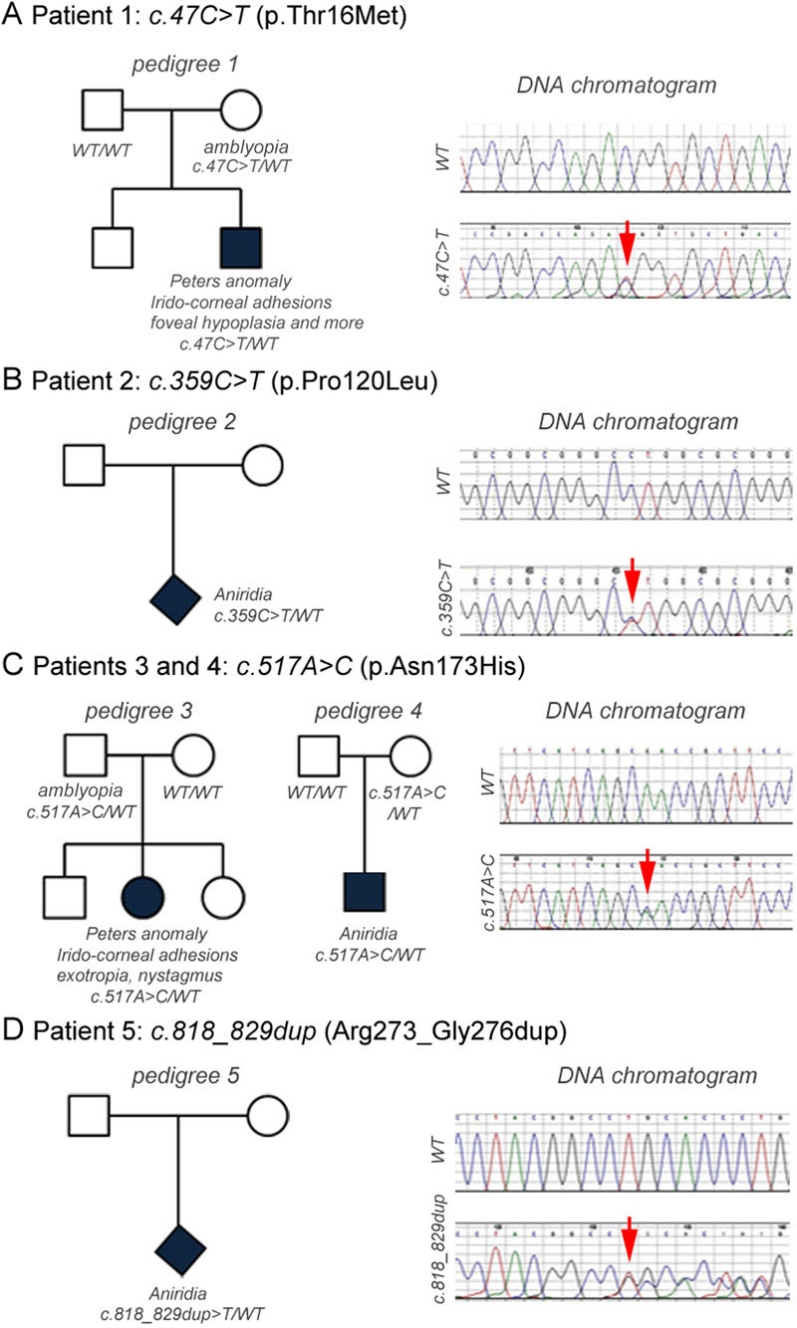
Identification of *FOXD3* sequence variants in Peters anomaly and aniridia. Pedigrees and sequence chromatograms for variants identified in Patients 1–5 (**A-D**). The mutations are indicated (red arrows).

**Figure 2:**
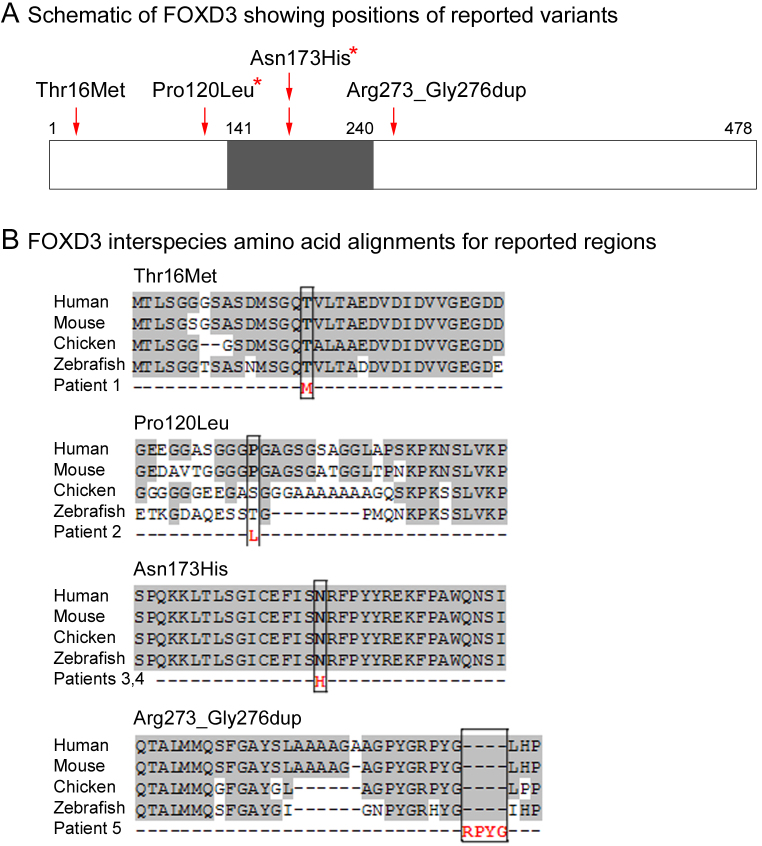
Position and interspecies conservation of FOXD3 variants. Schematic of the FOXD3 protein showing the forkhead domain (shaded) and positions of the amino acid variants affecting conserved regions; variants that showed a statistically significant association with the patient population (see text) are marked with an asterisk (**A**). Amino acid alignments of vertebrate FOXD3 proteins for the corresponding regions (**B**).

The first variant, c.47C>T, is predicted to cause a p.Thr16Met substitution in the NH_2_-terminus of the FOXD3 protein (Table 1, Figure 1, and Figure 2). This heterozygous variant was seen in Patient 1, a six-month-old Caucasian male with left Peters anomaly with iridocorneal adhesions, right congenital cataract with anterior synechiae, bilateral iris and lens colobomas, abnormal iris and corneal vascularization, persistent hyperplastic primary vitreous, and foveal hypoplasia. There were no systemic anomalies. The mother was reported to have had a ‘lazy eye’ as a child, which was treated by patching, and currently has moderate anisometropia. The patient’s father and brother are unaffected. The mother also carried the p.Thr16Met allele (Figure 1). The methionine substitution affects a highly conserved domain near the NH_2_-terminus of FOXD3 and the position is occupied by threonine in all known vertebrate orthologs (Figure 2). The p.Thr16Met variant was not observed in 456 in-house controls, but is heterozygous in 8 of 5,276 individuals in the EVS. The frequency of the p.Thr16Met allele shows no significant difference between cases and controls based on Fisher’s exact test (1/232 for Peters anomaly/aniridia versus 8/11464 control chromosomes, p=0.165).

The second variant, c.359C>T, is predicted to cause a proline to leucine substitution at position 120 in the NH_2_-terminal region of FOXD3, 18 residues before the forkhead domain (Table 1, Figure 1 and Figure 2). This variant was detected in Patient 2 with aniridia. The gender, ethnicity and family history are not known. The substitution affects a region with moderate evolutionary conservation; the proline at this position is conserved among mammals, but it is replaced by serine and tyrosine in chick and zebrafish orthologs, respectively (Figure 2). The p.Pro120Leu variant was observed in 1 sample among 455 in-house controls, and was not observed among the EVS individuals (n=4,489). Based on Fisher’s exact test, the frequency of this variant is statistically increased in aniridia and Peters anomaly cases (1/232 versus 1/9888 chromosomes, p=0.045).

The third variant, c.517A>C, is predicted to cause a p.Asn173His substitution within the forkhead domain (Table 1, Figure 1, and Figure 2) and was identified in two unrelated patients in our cohort. Patient 3 is a 10-year-old Caucasian female with unilateral Peters anomaly (iridocorneal adhesion), exotropia, and horizontal nystagmus. She also has a mild hearing deficit in the left ear, minimally posteriorly rotated ears (left>right), a broad nasal bridge, and mild clinodactyly. The patient’s mother is unaffected, while her father was reported to have had a ‘lazy eye’ treated by patching as a child, but no other clinical details were available. Patient 3 has one brother and one sister, who were reported to be unaffected but were unavailable for screening. The father also carries the p.Asn173His change (Figure 1). Patient 4 is a 10-year-old Caucasian male with aniridia. Both of his parents are reported to be unaffected and there are no siblings. The patient’s mother also carries the p.Asn173His allele (Figure 1). The substitution affects an amino acid in the forkhead domain that is 100% conserved among vertebrate FOXD3 orthologs (Figure 2). The p.Asn173His variant was not observed in 473 in-house controls but was detected 1 of 5,379 individuals in the EVS. The p.Asn173His allele frequency is statistically increased in aniridia and Peters anomaly cases (2/240 versus 1/11,704 control chromosomes, p=0.001).

The fourth variant is a 12-base-pair in-frame duplication, c.818_829dup, which is predicted to cause a tandem duplication (p.Arg273_Gly276dup) of four amino acids (Arg-Pro-Tyr-Gly) near the COOH-terminus, downstream of the forkhead domain (Table 1, Figure 1 and Figure 2). This was detected in Patient 5 with aniridia. The gender, ethnicity, and family history are not known. The duplication affects a moderately conserved region of FOXD3. The p.Arg273_Gly276dup variant was not observed in 455 in-house controls or the SNP database (insertions and deletions are not recorded in the EVS so the presence/absence of the duplication in this population could not be determined). Interestingly, the c.818_829dup duplication was also identified in two additional unrelated ocular patients in our cohort, a Caucasian male with isolated anophthalmia and a female with isolated bilateral congenital cataracts. Even though this variant was not found in any control samples in our study or the SNP database, the difference was not statistically significant based on Fisher’s exact test (1/238 versus 0/910 chromosomes, p=0.207 for aniridia/ Peters anomaly patients; 3/610 versus 0/910 chromosomes, p=0.064 for all ocular cases).

Two other nonsynonymous variants identified included c.262_273del (p.Ala88_Gly91del) and c.286G>T (p.Val96Leu; Table 1). Both of these variants affect nonconserved regions and residues, and were observed at a similar frequency in cases and controls with p values of 1 and 0.597, respectively.

Since *PAX6* mutations account for the majority of aniridia cases, and have also been reported in Peters anomaly, iridocorneal adhesion and foveal hypoplasia phenotypes [31,32], we carefully examined the *PAX6* coding region and copy number in Patients 1–5, and found no alterations (data not shown).

## Discussion

*FOXD3* plays an important role in the specification and development of the neural crest cells that make a significant contribution to human ocular structures. Analysis of *FOXD3* mutations in human ocular disease is the first step toward characterizing its potential involvement in human eye development and pathology. Here, we report four *FOXD3* variants identified primarily in patients with Peters anomaly and aniridia and affecting conserved regions of the FOXD3 protein. Peters anomaly is a developmental defect characterized by central corneal opacity, defects in the posterior layers of the cornea, and variable lenticulo-corneal and irido-corneal adhesions [38,^39^]; aniridia is a panocular disorder in which the iris is absent or severely reduced, with additional findings including corneal opacification, glaucoma, cataracts and foveal hypoplasia [31,32,40]. No enrichment for *FOXD3* variants was observed in other ocular phenotypes: while the p.Arg273_Gly276dup mutation was detected in one case each of anophthalmia and congenital cataracts, no other significant variants in *FOXD3* were identified in the sizable population screened with these phenotypes (n=123).

In all five Peters anomaly/aniridia families reported here, the clinical phenotype was only noted in the proband and mutations in *PAX6,* the primary aniridia gene [31,32] were excluded; in two families, the parent carrying the *FOXD3* variant had a ‘lazy eye’ (ambylopia) during childhood. All four variants involve conserved positions in the FOXD3 protein; variants p.Thr16Met and p.Asn173His alter residues that are 100% identical among vertebrates. Two variants, p.Asn173His and p.Pro120Leu, showed a statistically significant increase in allele frequency in the Peters anomaly/aniridia subgroup in comparison to controls and one allele, p.Arg273_Gly276dup, was not detected in our control samples or reported by others, but was found in two additional ocular cases with anophthalmia and cataract phenotypes in this study.

The p.Asn173His substitution is of particular interest since it was observed in two unrelated probands with Peters anomaly/aniridia and affects the DNA-binding domain. The presence of this variant in one normal control chromosome (out of 11,702) is consistent with the reduced penetrance, or subclinical expressivity, seen in the unaffected carrier parents. Similarly, incomplete penetrance and variable expressivity have been observed in families with heterozygous mutations in orthodenticle homeobox 2 (*OTX2*) [41] or bone morphogenetic protein 4 (*BMP4*) [42], which are associated with anophthalmia/microphthalmia or severe anterior segment dysgenesis.

Additional studies are required to identify the functional consequences of the reported variations. The p.Thr16Met, p.Pro120Leu and p.Arg273_Gly276dup variants involve NH_2_- or COOH-terminal regions of FOXD3 which have not yet been assigned any specific function but are, in general, believed to play a role in the modulation of FOXD3 activity in different tissues/processes through interactions with other proteins. The p.Asn173His variant involves the forkhead domain of FOXD3 which is responsible for its interaction with DNA. Although asparagine 173 is not predicted to contact DNA, it may interact with other proteins, modulating DNA binding affinity/specificity through allosteric effects. The NMR structure of the rat Foxd3/Genesis forkhead domain has been determined, both in the free state and in a Foxd3-DNA complex [43-45]. In the unbound state, the FOXD3 forkhead domain contains four α helices, three β strands, and two wing motifs. The fourth helix (H4) is unique to FOXD3. It is located between helices 2 (H2) and 3 (H3), and replaces a random coil segment in other FOX proteins. Upon DNA binding, FOXD3 undergoes a conformational change that perturbs the wing domains, decreases overall flexibility, and induces a fifth helical fold (H5), which is absent in the free state [43]. The junction between H2 and H3 is thought to play a role in DNA-binding specificity [46]. H3 makes direct contact with the DNA major groove, while wing domain W2 interacts with the minor groove [46,47]. The asparagine 173 is located near the carboxyl end of helix 2 (H2) in the forkhead DNA-binding domain. The p.Asn173His substitution introduces an ionizable and possibly positively charged side chain to this region. Though the exact consequences of this change are unknown, the p.Asn173His substitution may disrupt a protein interaction or alternatively recruit an erroneous protein to the site. These effects may be tissue specific and require the presence of particular cofactors that are yet to be determined.

Several previously reported mutations affect the H2 helix of other forkhead proteins. The recessive mouse mutation *dyl* (dysgenic lens) results from two concurrent amino acid substitutions in the *Foxe3* gene, p.Phe93Leu and p.Phe98Ser. Mice homozygous for both mutations have small eyes with a fused lens and cornea, absent secondary lens fibers, and cataracts [24]. Some heterozygous *dyl^-^*^/+^ mice (40%) have a milder corneal and lens phenotype, including corneal opacity, keratolenticular adhesions and cataracts [25]. Phe93 is located in helix H2, and Phe98 is located in the interhelical region between H2 and H3. In human *FOXC1*, a phenylalanine–to-serine substitution (p.Phe112Ser) in the first residue after helix H2 was discovered in a large family with heterogeneous anterior segment defects [26]. The mutation decreased transcriptional activation in an in vitro cotransfection assay, but did not detectably impair DNA-binding [27].

In summary, we report the identification of four *FOXD3* variants in five human patients with the anterior segment dysgenesis phenotypes Peters anomaly and aniridia. These findings are consistent with the role of *Foxd3* in specification of the neural crest emerging from animal model studies [10,13-15]. Although enriched in the Peters anomaly/aniridia cohort, the presence of these rare *FOXD3* variants in unaffected family members and controls suggests that additional genetic, environmental or stochastic factors may be required for expression of the disease phenotype. The secondary factor(s) may involve proteins that interact directly with FOXD3 or modulate its activity. Future biochemical and genetic studies of the FOXD3 pathway may identify cofactors as new candidate genes for eye disease and clarify the contribution of *FOXD3* mutations to human eye malformations.

## Appendix 1. Oligonucleotides used for PCR and sequencing of the *FOXD3* and *PAX6* genes.

To access the data, click or select the words “Appendix 1.” This will initiate the download of a compressed (pdf) archive that contains the file.
